# Sex-specific responses to cold in a very cold-tolerant, northern *Drosophila* species

**DOI:** 10.1038/s41437-020-00398-2

**Published:** 2021-01-28

**Authors:** Darren J. Parker, Tapio Envall, Michael G. Ritchie, Maaria Kankare

**Affiliations:** 1 Department of Ecology and Evolution, University of Lausanne, Lausanne, Switzerland; 2 Swiss Institute of Bioinformatics, Lausanne, Switzerland; 3 Department of Biological and Environmental Science, University of Jyväskylä, Jyväskylä, Finland; 4 Center for Biological Diversity, School of Biology, University of St. Andrews, Fife, Scotland UK

**Keywords:** Evolutionary genetics, Gene expression

## Abstract

Organisms can plastically alter resource allocation in response to changing environmental factors. For example, in harsh conditions, organisms are expected to shift investment from reproduction toward survival; however, the factors and mechanisms that govern the magnitude of such shifts are relatively poorly studied. Here we compared the impact of cold on males and females of the highly cold-tolerant species *Drosophila montana* at the phenotypic and transcriptomic levels. Although both sexes showed similar changes in cold tolerance and gene expression in response to cold treatment, indicating that the majority of changes are concordant between the sexes, we identified a clear reduction in sexually dimorphic gene expression, suggesting that preparing for the colder season involves reducing investment in sex-specific traits. This reduction was larger in males than females, as expected if male sexual traits are more condition-dependent than female traits, as predicted by theory. Gene expression changes were primarily associated with shifts in metabolic profile, which likely play a role in increasing cold tolerance. Finally, we found that the expression of immune genes was reduced following cold treatment, suggesting that reduced investment in costly immune function may be important in helping flies survive colder periods.

## Introduction

Life history strategies involve strategic allocation of investment between reproduction and survival, and relative investment in these depends on a wide range of intrinsic and extrinsic factors (Stearns 1992, 2000; Roff 2002). Many of these factors (e.g., age, density, and reproductive status) vary throughout an organism’s lifetime, meaning selection will favor different allocations at different times or under different conditions. As a result, organisms are typically able to plastically shift the relative allocation of resources in response to environmental cues, particularly when changes in the environment are predictable (Schlichting 1986; Scheiner 1993).

One predictable shift is the change from summer to winter, when temperature is decreasing and day length is shortening. For organisms at high latitudes, this harshening of the environment is expected to produce a shift in resource allocation with a greater investment into survival over reproduction. This is because, in order to survive the colder conditions, organisms need to produce a range of costly metabolites and proteins and to begin to store resources to survive the colder season (Lee 1991; Denlinger and Lee 2010). Numerous factors are likely to influence the magnitude of these trade-offs, including life cycle, age, and condition. One potentially important factor, which is however surprisingly rarely studied, is that of sex. With the changing of the seasons, both males and females have to adjust to the same conditions, so we expect that the physiological shifts to survive colder temperatures may be similar. However, the relative costs of coping with lower temperatures may differ between the sexes. For instance, males may be more susceptible to cold than females (e.g., in *Drosophila* (*D*.) *melanogaster*; Bubliy et al. 2002) meaning that a greater shift in resources would be required in order for males to survive colder periods. In addition, although sexual traits in both sexes are expected to be reduced in response to worsening conditions (Andersson 1982; Rowe and Houle 1996; Cotton et al. 2004; Bonduriansky 2007), it is also expected that condition dependence will be stronger for males than females due to sexual selection (Trivers 1972; Kokko 2001), meaning we could expect a larger shift in resources in males than in females when they prepare for the onset of cold. Finally, males and females typically have very different gene expression profiles, expressing a large fraction of genes at different levels throughout the genome to produce sexually dimorphic phenotypes (Grath and Parsch 2016; Mank 2017). Such sex differences in gene expression profile could restrict additional changes in gene expression. For instance, if increased expression of a gene would have negative effects in one sex but not the other (Innocenti and Morrow 2010) or if a gene is already maximally expressed in one sex but not the other, then this could produce differences in how each sex can respond to cold.

Here we use a cold treatment to examine how *Drosophila* (*D*.) *montana* responds to the onset of cold. This species is distributed at high latitudes (30–70°N) across North America, Asia, and Europe (Throckmorton 1982) and is the most cold-tolerant *Drosophila* species studied (Kellermann et al. 2012a; Vesala et al. 2012). As such, this species is particularly well adapted to cold environments (Vesala et al. 2012; Kellermann et al. 2012b) with both sexes able to overwinter at subzero temperatures as adults, meaning that being able to survive cold stress is an important part of their life history. We have three objectives. First, we examine if males and females have similar phenotypic responses to the onset of cold. Second, we examine if males and females have similar changes in gene expression when subjected to cold using an RNA-seq approach. This is ideal for examining how shifts in resource allocation occur since these changes are plastic, and thus differences in gene expression will reflect differences in resource allocation strategies. We predict that (1) males and females will show similar phenotypic changes to cold, (2) both sexes will show similar changes in gene expression for most genes, (3) genes associated with producing sexual differences in traits (i.e., sex-biased genes) will show significant reductions in expression in response to cold, and (4) this reduction will be larger in males than in females. Finally, we examine the functional processes associated with genes that change expression to gain insight into the molecular mechanisms by which males and females cope with the onset of cold.

## Materials and methods

### Samples

A genetically variable population cage was established using twenty fertilized *D. montana* females collected in 2013 from Korpilahti (62°N), Finland. This population cage was maintained in constant light at 19 °C to prevent the flies from entering diapause, but note that *D. montana* does not lose circadian clock rhythmicity in constant light, in contrast to *D. melanogaster* (Kauranen et al. 2012). To ensure that all flies were of the same age, newly enclosed flies from the cage population were anesthetized with CO_2_ and separated by sex under a microscope, and separate sexes were placed into half-pint bottles with yeast–malt medium (~20 flies were kept in each bottle). For the next 16 days, the bottles were kept at 19 °C, and flies were transferred to new bottles every week. After 16 days, half of the virgin females and males were subjected to the cold treatment, which was 5 days at 6 °C (Vesala et al. 2012) and the remainder of the flies served as a control group remaining at 19 °C. At 21 days (when flies are sexually mature; Salminen and Hoikkala 2013), we performed phenotyping and RNA extractions. Note that individuals were taken randomly from replicate bottles and different individuals were used for phenotyping and RNA extractions.

### Phenotypic measurements of cold tolerance

The phenotypic effect of cold treatment on cold tolerance was determined by measuring the critical thermal minimum (CTmin) of the flies. CTmin is the temperature at which flies lose neuromuscular function, causing them to fall into a reversibly immobilized state called chill–coma (see Andersen et al. 2015 for details). In all, 21-day-old flies were put into 10-cm-long glass tubes of diameter 1 cm, with 2–3 flies per tube. Flies in tubes were kept apart by pieces of plastic foam. The tubes were then sealed with Parafilm and submerged into a 30% glycol–water mixture within a Julabo F32-HL refrigerated/heating circulator. Temperature of the liquid was then decreased at the rate of 0.5 °C/min from 19 to –10 °C for all individuals as to be slow enough to allow the insect’s body temperature to cool with the temperature in the chamber, but fast enough to avoid a substantial physiological response during the cooling (Sinclair et al. 2015).

CTmin was recorded as the temperature at which a fly entered a chill–coma state by falling down. The experiment was conducted in batches of no more than 8 tubes with a maximum of 24 flies for a total of 137 flies (32–37 flies per treatment). Flies were cooled to a point at least a couple of degrees celsius below the temperature at which the last fly had entered chill–coma. Afterward, the tubes were incubated at room temperature until all the flies had recovered from the chill–coma, to make sure that all the flies were normal, healthy individuals. Finally, the flies were killed by putting them into a freezer (–20 °C) for at least 12 h, after which their weight was measured. The effects of temperature, sex, temperature-by-sex interaction, and weight on (log-transformed) CTmin were then tested using a generalized linear mixed model in the lme4 package (v. 1.1.14) (Bates et al. 2015) in R (v. 3.5.1) (R Core Team 2017). Temperature, sex, temperature-by-sex interaction, and weight were fit as fixed effects, with batch included as a random effect. Statistical significance of fixed effects was determined using a Wald test. Note that a value of five was added to all values of CTmin before log-transforming data to avoid taking the log of negative values.

### RNA extraction and sequencing

In all, 21-day-old flies were collected from the maintenance chambers and flash-frozen in liquid nitrogen and pooled into 12 samples with three flies in each sample for both cold-treated and control groups. Flies were crushed with a plastic mortar, after which RNA extraction using ZR Insect & Tissue RNA Micro Kit with DNase treatment (Zymo Research) was carried out. RNA concentration was measured with Qubit (ThermoFisher), purity with NanoDrop ND-1000 (NanoDrop Technologies), and integrity with TapeStation 2200 (Agilent Technologies). Strand-specific library preparation (one library per sample) and paired-end (150 + 150 bp) Illumina sequencing (Illumina HiSeq 3000, 5 lanes) was then performed at the Finnish Functional Genomics Center, Turku, Finland.

### Read trimming and mapping

Raw reads were trimmed before mapping. First, CutAdapt (Martin 2011) was used to trim adapter sequences from the reads before further trimming reads using Trimmomatic v 0.36 (Bolger et al. 2014). All reads were trimmed to 140 bp, then quality-trimmed with the following options: LEADING:30 TRAILING:30 SLIDINGWINDOW:17:19. Any reads <85 bp in length after trimming were discarded. Quality-trimmed reads from each library were then mapped separately to the *D. montana* reference genome (Accession Number: LUVX00000000.1) (Parker et al. 2018) using STAR (v. 2.4.2a) (Dobin et al. 2013) with default options. Read counts for each gene were then obtained using HTSeq (v. 0.9.1) (Anders et al. 2015).

### Differential gene expression analysis

Expression analyses were performed using the Bioconductor package EdgeR (v. 3.24.0) (Robinson et al. 2010) in R (v. 3.5.1) (R Core Team 2017). Genes with counts per million < 0.5 in two or more libraries per condition were excluded. Normalization factors for each library were computed using the TMM method. To estimate dispersion, we fit a generalized linear model with a negative binomial distribution with the terms sex, temperature, and their interaction. A quasi-F test was used to determine the significance of model terms from this model for each gene by comparing appropriate model contrasts, with *p* values corrected for multiple tests using Benjamini and Hochberg’s (1995) algorithm. Statistical significance was set to 5%. Whether genes differentially expressed in males and females showed a greater overlap than expected by chance was determined using the SuperExactTest package (v. 0.99.4) (Wang et al. 2015).

Sex-biased genes were classified as genes showing significant differences (FDR < 0.05, |log_2_FC|≥1) between males and females in both control and cold-treated samples. Genes found to be sex-biased in control and cold-treated samples showed good agreement (Fig. S1 and Table S1). We chose these thresholds in order to select a robust set of sex-biased genes and to reduce the effect of sex-biased allometry (Montgomery and Mank 2016). To test if the expression of sex-biased genes changes in response to cold, we used a Wilcoxon test corrected for multiple tests using Benjamini and Hochberg’s algorithm. To compare the magnitude of the shift in sex-biased gene changes in males and females, we normalized the changes in gene expression in sex-biased genes by the median change observed in unbiased genes, and then calculated the pseudomedians and 95% confidence intervals of the change using the wilcox.test function in R. We also repeated this analysis when sex-biased genes were defined with the additional condition that they are also sex-biased in a close relative of *D. montana*, *Drosophila* (*D.*) *virilis*. Values for sex-biased expression in *D. virilis* were obtained from the sex-associated gene database (Shi et al. 2019) (downloaded November 5, 2019). Genes sex-biased in *D. montana* and *D. virilis* showed good agreement (Fig. S2). To examine the overall similarity of male and female gene expression in each condition, we compared Spearman’s correlation coefficients of male and female gene expression (as mean log_2_ CPM) in control and cold-treated flies using a Fisher’s *z*-test implemented in the cocor package (Diedenhofen and Musch 2015) in R (R Core Team 2017).

### Functional annotation clustering

Functional annotation clustering of differentially expressed genes was carried out using DAVID (Database for Annotation, Visualization, and Integrated Discovery) v. 6.8 (Huang et al. 2009a, b) with *D. melanogaster* orthologs (7698, obtained from www.flymine.org). When multiple orthologs were obtained (144 genes), one was chosen at random to be used in DAVID. DAVID clusters genes into functional groups using a “fuzzy” clustering algorithm, and then uses a Fisher’s exact test to identify significantly enriched functional groups. A functional group was considered to be significantly enriched if its enrichment score was >1.3 (*p* < 0.05).

## Results

### Males and females show a similar phenotypic response to cold

**Fig. 1:**
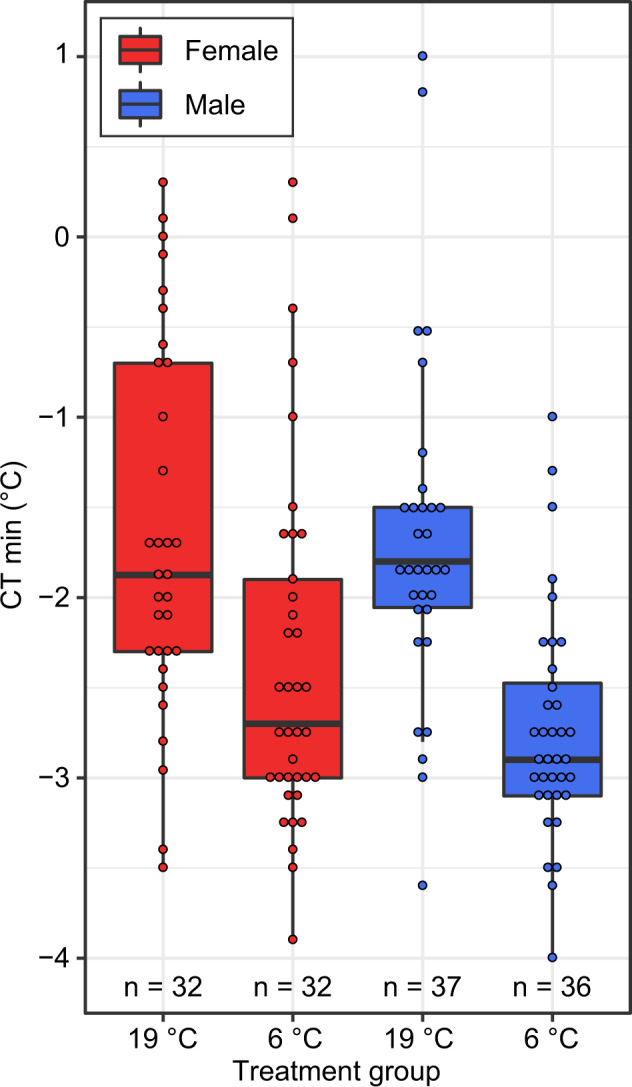
Cold tolerance is higher for males and females when maintained at a colder temperature. Treatment group indicates whether flies were maintained at 19 or 6 °C for 5 days (see text for detailed methods). CTmin is the temperature at which flies lose neuromuscular function.

To assess if males and females have a similar response to cold, we experimentally reduced the temperature flies were maintained at from 19 to 6 °C, representing average daytime temperatures in Central Finland in late July and early October, respectively (www.worlddata.info). After 5 days, we compared the CTmin (the temperature at which flies lose neuromuscular function) of cold-treated flies to control flies (see methods for details). Both males and females showed a significant increase in cold tolerance (i.e., a lower CTmin value following cold treatment, Fig. 1 and Table 1). There was no significant treatment by sex interaction, indicating that males and females have a similar phenotypic shift in cold tolerance (Table 1).

**Table 1:** The effect of sex, cold treatment, and weight assessed by a generalized linear mixed model (full model).

Model term	Wald chi-square	*p*
Sex	0.0391	0.40883
Cold treatment	11.1732	**0.02637**
Weight	0.8657	0.64130
Sex × cold treatment	1.39	0.93485

Significant *p* values are in bold.

### Males and females show similar changes in gene expression in response to cold

**Fig. 2:**
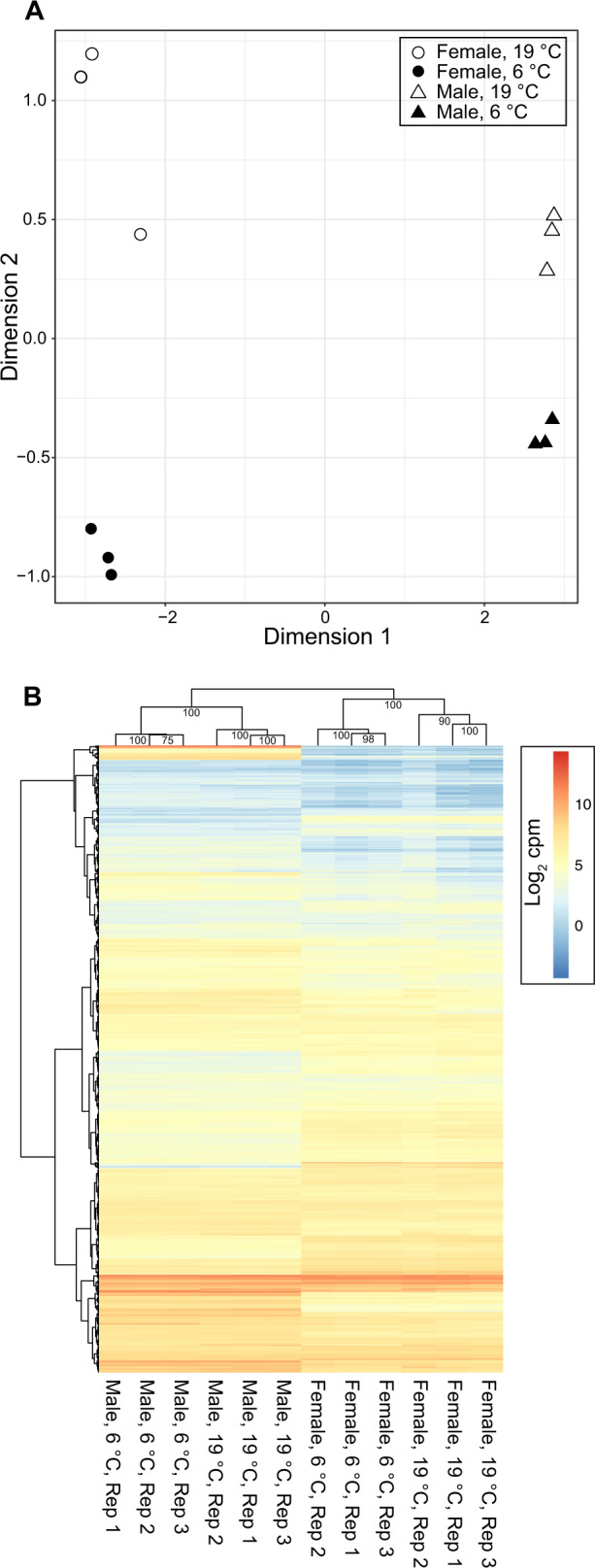
Samples cluster by cold treatment and sex. **A** MDS plot of male (triangle) and female (circle) expression when kept at 19°C (empty shapes) or 6°C for 5 days (filled shapes). Distances between samples in the MDS plot approximate the log_2_ fold change of the 500 genes with the largest biological variation between the libraries. **B** Heatmaps and hierarchical clustering of gene expression (log_2_ CPM). Values on each node show the bootstrap support from 1000 replicates.

**Fig. 3:**
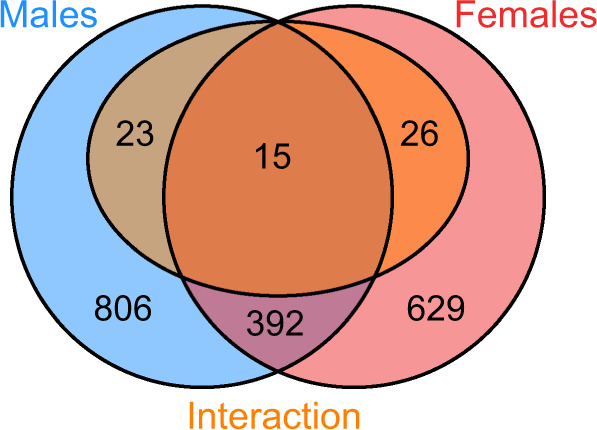
Venn diagrams showing the overlap of genes differentially expressed in response to cold treatment in males (blue) and females (red). Genes with a significant sex-by-treatment interaction are shown in orange, showing that the majority of genes differentially expressed in males and females do not have significant sex-by-treatment interactions (color figure online).

**Fig. 4:**
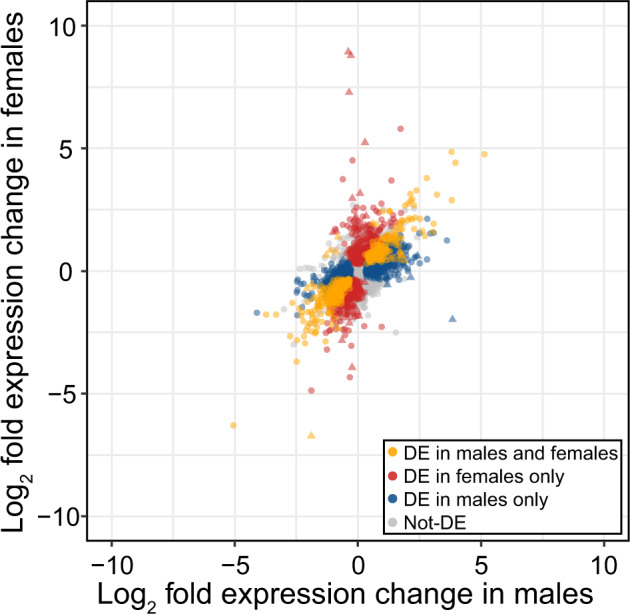
Expression change in males and females in response to cold treatment, indicating genes differentially expressed in males and females (orange), females only (red), males only (blue), and in neither sex (gray). Triangle points indicate a significant sex-by-treatment effect (color figure online).

Flies raised under the same conditions used for phenotypic measurements (above) were also used for gene expression analyses. Both sex and temperature strongly influenced gene expression (Fig. 2A) with samples clustering first by sex, then by temperature (Fig. 2B). Differential expression analyses found that a little over 10% of all expressed genes were differentially expressed in response to cold in both males (1236/9338) and females (1062/9338), with significant overlap between genes differentially expressed in males and females (Fig. 3). This overlap was much greater than expected by chance (*p* = 1.7 × 10^–110^). Gene expression change in response to cold was also highly correlated between males and females for all genes (rho = 0.59, *p* value < 2.2 × 10^–16^, Fig. 4), and for genes differentially expressed in either males or females (rho = 0.73, *p* value < 2.2 × 10^–16^) with only a small number of genes (64) showing a significant sex-by-temperature interaction (Figs. 3 and S3).

### Sexually dimorphic gene expression is reduced in response to cold

**Fig. 5:**
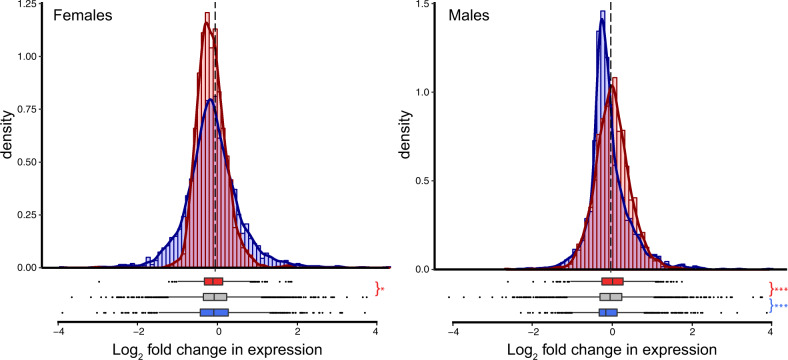
Expression shifts in sex-biased genes following cold treatment in females and males. Positive values indicate increased expression in cold-treated flies. Asterisks indicate the significance level (FDR) of Wilcoxon tests comparing the change in expression in female-biased (red, *N* = 1682) and male-biased (blue, *N* = 2023) genes to unbiased genes (***<0.001, **<0.01, *<0.05) (color figure online).

**Fig. 6:**
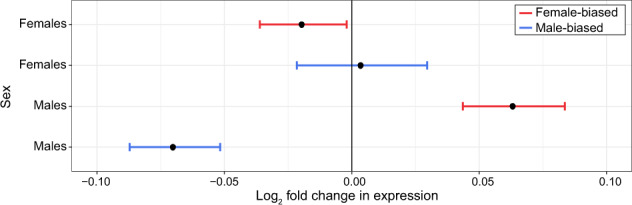
Expression shifts in female-biased (red, *N* = 1682) and male-biased (blue, *N* = 2023) genes following cold treatment in females and males relative to the median expression of unbiased genes. Positive values indicate increased expression in cold-treated flies. Points indicate pseudomedian and error bars indicate the 95% confidence interval (color figure online).

To determine if exposure to cold alters the amount of sexually dimorphic gene expression, we first examined how sex-biased genes change in response to cold. We found that the amount of sexually dimorphic gene expression decreased in both males and females. More specifically, we found that male-biased genes reduced in expression and female-biased genes increased in expression in males, and female-biased genes decreased in expression in response to cold in females (Fig. 5). Shifts in sex-biased genes were larger in males than females, for both male- and female-biased genes, leading to a more “feminized” transcriptome overall (Fig. 6). Note that similar shifts in sex-biased gene expression were also found when using a more conservative set of sex-biased genes (genes that are sex-biased in both *D. montana* and *D. virilis*; Figs. S2 and S4); however, changes in expression in females were no longer significant. Next, we examined the correlation of gene expression for males and females for all genes in each temperature treatment. Correlations between male and female gene expression were significantly higher for flies kept at 6 °C than those at 19 °C (Fisher’s *z*-test, *p* = 0.0163), though the magnitude of this difference was small (19 °C *r* = 0.686, 6 °C *r* = 0.704).

### Genes differentially expressed in response to cold are enriched for metabolism and immune response in males and females

We used functional annotation clustering to examine the function of genes differentially expressed in response to cold. Specifically, we used this approach to identify processes enriched for genes differentially expressed in both males and females, genes differentially expressed in males only, genes differentially expressed in females only, and genes showing a sex-by-treatment interaction (Table S2). The largest number of enriched gene clusters was found from genes differentially expressed in both males and females. These were primarily connected to metabolism (e.g., lipid metabolism, fatty acid biosynthesis, carbohydrate kinases, metalloproteases, and aminotransferases) as well as to the immune response (e.g., innate immune response and DM9 repeat), suggesting that these processes are important for both males and females to adjust to a colder environment. Interestingly, all genes in immune-related clusters showed decreased expression following cold treatment (Fig. S5), suggesting a reduced investment in immune function. Five other innate immune response (GO:0045087) genes were differentially expressed in response to cold in males only (*Toll-7* and *Dredd*) or females only (*PGRP-SB1*, *Src42A*, and *pll*). None of these genes showed a significant sex-by-treatment interaction, suggesting that changes in immune response are largely concordant between the sexes. In contrast to the innate immune response genes differentially expressed in response to cold in both sexes, two of the five genes differentially expressed in only one sex are upregulated in response to cold (*Toll-7* and *Src42A*); however, both of these genes are also involved in a number of other processes. Genes showing a significant sex-by-treatment interaction were enriched for transmembrane transport, suggesting that while changes to this process are important for both males and females in a colder environment, how it is mediated differs between the sexes (Fig. S6). Finally, we found that the functional clusters enriched in genes differentially expressed only in females or only in males were different. In females, clusters were related to oxidoreductase activity and the biosynthesis of amino acids, whereas in males, clusters were related to cytoplasmic translation, protein biosynthesis, transmembrane transport, ATP-binding domain, glutamine metabolic processes, and nucleotide binding (Table S2).

## Discussion

In order to survive harsh conditions, organisms are expected to shift investment toward survival and away from reproduction. This shift may differ between the sexes due to relative differences in the costs of reproduction or survival, or because of differences in regulatory architecture (Grath and Parsch 2016; Mank 2017). Despite its importance, the underlying mechanisms responsible for shifts in investment are poorly studied (Flatt and Heyland 2011). Here we examined such a shift by investigating the phenotypic and transcriptomic changes associated with the onset of cold in males and females of a cold-tolerant fly species, *D. montana*.

We found that both sexes show a similar change in phenotype following cold treatment. Changes in cold tolerance (measured by CTmin) might be expected to be similar as both males and females have similar baseline cold tolerances in benign temperatures (Kellermann et al. 2012a) and because both sexes need to adjust their physiology in order to survive in colder temperatures. However, although cold tolerances are similar at benign temperatures, this need not be the case. For instance, in the more temperate, but closely related species, *D. virilis*, males show much lower cold tolerances than females at benign temperatures (Kellermann et al. 2012a). Why this is not the case in *D. montana* is unclear, but one possibility is that *D. montana’s* ability to survive extremely cold temperatures constrains cold tolerance at warmer temperatures in both sexes.

Changes in gene expression in response to cold were similar between the sexes, suggesting that males and females adjust their physiology using largely the same mechanisms. Unfortunately, very few studies have examined gene expression changes in males and females in response to stressful environmental conditions; however, our results agree with a previous study showing that most genes in male and female *D. melanogaster* are regulated similarly in response to changes in dietary composition (Camus et al. 2019). While we found that most gene expression differences were similar between the sexes, it is clear that there are also differences. We observed a reduction in sexually dimorphic gene expression, with males and females having more similar expression in the harsher, colder condition. This agrees with previous studies in *D. melanogaster* (Wyman et al. 2010) and beetles (Kijimoto et al. 2014; Ledón-Rettig and Moczek 2016; Zinna et al. 2018), which showed a reduction of sexually dimorphic gene expression with reduced environmental quality. The shifts in sex-biased gene expression we observed were relatively small overall, but it is notable that the shifts were larger in males than females. This is likely because investment into sexual traits is more condition-dependent in males than females (Trivers 1972; Kokko 2001), suggesting that a reduction of investment into expensive male functions during winter represents a greater change in life history than female changes (see also Wyman et al. 2010; Malacrinò et al. 2019). How changes in sex-biased gene expression are mediated is not fully understood; however, one potential mechanism is by a change in expression of the key transcription factors involved in sexual differentiation, *doublesex* (*dsx*) and *fruitless* (*fru*) (Neville et al. 2014; Clough et al. 2014; Ledón-Rettig et al. 2017). Intriguingly, we find *dsx* (but not *fru*) expression increased in males in response to cold. Such a change could potentially mediate the changes in sex-biased gene expression we found; however, future work would be needed to confirm this.

Relatively few genes showed a significant sex-by-treatment interaction, reinforcing the idea that most changes in gene expression are concordant between the sexes. Interestingly however, these few genes were enriched for transmembrane transport, a process that has been previously associated with cold adaptation in a number of insects (Overgaard and MacMillan 2017), including *D. montana* females (Parker et al. 2015). Changes to transmembrane transport are thought to be particularly important for surviving cold temperatures by preventing a loss of cellular ionic balance (Heitler et al. 1977; Hochachka 1986; Kivivuori et al. 1990; Koštál et al. 2004). Our results suggest that, despite its importance, changes to transmembrane transport are mediated by different genes in each of the sexes. The reasons for this are not clear, but it is possible that these differences may arise from sex-specific genetic constraints.

Overall, we found that the transcriptomic response to cold is extensive, with several hundred genes showing differential expression. By examining the functional processes associated with these gene expression changes, we are able to gain insights into the mechanisms by which males and females cope with the onset of cold. We did this in two separate analyses, first examining processes enriched in genes differentially expressed in both sexes, then processes enriched in genes differentially expressed only in males or only in females. Processes enriched in both sexes included many that have been previously associated with increasing cold tolerance, including metabolic shifts in lipids and carbohydrates. By altering their metabolic profile, insects are able to maintain osmotic balance and stabilize the membrane structures of a cell as temperatures decrease (e.g., Hazel 1995; Koštál et al. 2003; Overgaard et al. 2008; Andersen and Overgaard 2019). In particular, changes in lipids, fatty acids, and polyols have been shown to be important for cold adaptation in many insect taxa (Lee 1991; Michaud and Denlinger 2006; Kayukawa et al. 2007; Denlinger and Lee 2010), including *D. montana* females (Vesala et al. 2012; Parker et al. 2015; Vigoder et al. 2016). This is consistent with the changes we identify here, including the differential expression of previously identified candidate genes *Inos* and *CG6910* (Parker et al. 2015; Vigoder et al. 2016). Both of these genes belong to the inositol biosynthetic pathway, emphasizing the importance of this pathway for surviving colder temperatures in *D. montana*. Further support for the importance of fatty acids for surviving colder temperatures comes from examining the overlap between genes differentially expressed in males and females with genes associated with variation in population CTmin (Wiberg et al. 2020, see Fig. S7 and Table S3). This highlights three genes (*ORMDL*, *cue*, and *Fatp1*) each with roles in lipid homeostasis, including one (*Fatp1*), which has previously been shown to influence resistance to cold stress in *D. melanogaster* (Sujkowski et al. 2012).

Interestingly, we also found an enrichment of immune-related processes, including innate immune function and genes with a DM9 repeat (which likely have an antimicrobial function; Jiang et al. 2017; Liu et al. 2018). Genes enriched for these processes were reduced in expression following cold treatment, suggesting that investment in immune function is reduced in colder temperatures. This finding is in contrast to most previous work which shows that cold exposure stimulates an increase in immune function (e.g., *Ostrinia furnacalis* (Chen et al. 2019)*, Pyrrharctia isabella* (Marshall and Sinclair 2011), *Megachile rotundata* (Xu and James 2012; Torson et al. 2015), and *D. melanogaster* (Le Bourg et al. 2009; Zhang et al. 2011; MacMillan et al. 2016)). Such increases may represent a shift in investment for enhanced immune activity during colder periods; however, it is also consistent with a general stress response, or immune activation due to cold-induced tissue damage (MacMillan and Sinclair 2011). Since we observe a reduction in immune expression, the changes we see in immunity are unlikely to be as a result of general stress or tissue damage response, but instead due to a specific reduced investment in immunity. Interestingly, most of the immune genes downregulated in response to cold have key roles in antibacterial function, including *key* that specifically regulates the antibacterial immune response (Rutschmann et al. 2000), *PGRP-SD* that promotes the recognition of peptidoglycan (Iatsenko et al. 2016), and two *Attacin* genes that encode antimicrobial peptides effective against Gram-negative bacteria (Dushay et al. 2000; Wu et al. 2018). This suggests that a reduced investment in immunity may be specific to bacterial pathogens, implying that bacterial pathogens of *D. montana* are less prevalent or less virulent during cold periods (Ferguson et al. 2018). The only other study to our knowledge that found a reduction in immune function due to cold was performed with *Gryllus veletis* (Ferguson et al. 2016), which like *D. montana*, has an overwintering diapause stage (though as a nymph rather than an adult). Unlike other studies that used insects with an overwintering diapause stage (e.g., *Megachile rotundata*; Xu and James 2012), both our and Ferguson et al. (2016) study also used the developmental stage that will eventually enter into diapause (rather than developmental stages that cannot enter into diapause) for experiments. As such, the cold treatment used in our and Ferguson et al. (2016) study may represent the onset of seasonal cold and cueing these insects into preparing for a colder season, prior to entering into diapause. In these cases, reducing resource allocation in immunity is likely to be beneficial as maintaining immune function is energetically costly (Ardia et al. 2012) and insects need to conserve energy reserves to survive the colder temperatures (Williams et al. 2015; Sinclair 2015). As such, reducing investment in immune function may be a common adaptation for insects preparing for colder seasons, but future work in other species will be required to determine if this is a general phenomenon.

The processes enriched in genes differentially expressed in only one of the sexes are diverse. These processes represent changes that are potentially more important for one sex than the other; however, since few genes showed a significant sex-by-treatment interaction, many of these are also likely to have some role in both sexes. Although diverse, most of the enriched processes are involved in metabolism, including biosynthesis of amino acids and proteins, and glutamine metabolism, all of which have been previously associated with increased cold tolerance in insects (Clark and Worland 2008; Doucet et al. 2009; Denlinger and Lee 2010; Storey and Storey 2012; Teets and Denlinger 2013). In addition, we also found an enrichment of processes associated with oxidoreductase activity in females, which may help flies defend against increased oxidative stress induced by exposure to cold (MacMillan et al. 2016).

We interpret the differences we see in response to cold treatment as being involved in *D. montana’s* preparation for a colder season; however, since we only consider a short window of temperature change (5 days), it is possible that our experiment will miss some more seasonal changes. Future work looking at changes over a much longer time period (e.g., an entire season), along with the adjustment of other key environmental variables (e.g., light–dark cycle), are needed to gain a full understanding of the changes required for seasonal adaptation. In addition, although we expect the change in temperature to be responsible for the changes we see, it is possible that changing the temperature at which flies are maintained could affect other factors, such as the rate at which flies age, which could influence our results. We expect such changes to be minor, as the time flies were kept at different temperatures was relatively short, and because changes in gene expression due to aging are typically small (Jin et al. 2001; Lund et al. 2002). Finally, it is possible that mechanisms other than changes in gene expression are involved in producing the differences in cold tolerance we see (e.g., changes in the efficiency of neurotransmitter release or posttranscriptional modifications). While possible, we expect that the majority of changes involved in increasing cold tolerance will be due to changes in gene expression. This is because, in insects, increased cold tolerance mostly involves changing the cellular metabolic profile to maintain the osmotic balance and stabilize the cellular membrane structures (Hazel 1995; Koštál et al. 2003; Denlinger and Lee 2010). These changes require shifts in quantities and concentration of metabolic products that are likely to be produced through changes in gene expression.

In conclusion, we found that males and females respond to the onset of cold in a similar way at both the phenotypic and transcriptomic levels. Despite this, cold treatment also reduced the amount of sexually dimorphic gene expression, with males showing a larger reduction than females, suggesting that preparing for colder temperatures involves reducing investment in male-specific functions. Gene expression changes were mainly associated with shifts in metabolic processes; however, we also observed decreased expression of immune genes, suggesting that reduced investment in immunity may be an important adaptation to help survive the colder season. Finally, our results suggest that sex-specific adaptations involved in life history trade-offs are subtle but potentially important, even when they are not apparent at the phenotypic level, highlighting the importance of examining trade-offs at both phenotypic and molecular levels.

## Supplementary information

Supp figures and tables

Table_S1

Table_S5

Table_S6

## Data Availability

Raw reads have been deposited in SRA under accession codes SRR10960337–SRR10960348 (see Table S4). Scripts for the analyses in this paper are available at https://github.com/DarrenJParker/montana_sex-specific_responses_to_cold and are archived at Zenodo: 10.5281/zenodo.4322949. Raw CTmin data are given in Table S5. Full gene expression results are given in Table S6.
