# Supplementary material for: Sex-specific responses to cold in a very cold-tolerant, northern Drosophila species
Source: Heredity (Edinb). 2021 Jan 28;126(4):695–705. doi: 10.1038/s41437-020-00398-2 (PMC8182794; doi:10.1038/s41437-020-00398-2)
Supplement: Supplementary file 1 — Supp figures and tables [file 41437_2020_398_MOESM1_ESM.docx]

**Table S2 | Enriched functional processes**

| **Name** | **p** | **Sex** | **Description** |
| --- | --- | --- | --- |
| B1 | 0.002 | Both sexes | Lipid metabolism, fatty acid biosynthesis, fatty acyl-CoA synthase |
| B2 | 0.003 | Both sexes | DM9 repeat |
| B3 | 0.005 | Both sexes | Keto-sugar kinase, carbohydrate kinase |
| B4 | 0.010 | Both sexes | Flavin adenine dinucleotide binding, flavoprotein |
| B5 | 0.011 | Both sexes | Carboxypeptidase, metalloprotease |
| B6 | 0.014 | Both sexes | Pyridoxal phosphate-dependent transferase |
| B7 | 0.014 | Both sexes | Signal peptide, secreted, disulfide bond |
| B8 | 0.022 | Both sexes | Carboxypeptidase, metallocarboxypeptidase activity |
| B9 | 0.031 | Both sexes | Wax biosynthetic process, fatty acyl-CoA reductase, long-chain fatty-acyl-CoA metabolic process |
| B10 | 0.040 | Both sexes | Oxidoreductase activity, D-isomer specific 2-hydroxyacid dehydrogenase, NAD-binding, catalytic domain |
| B11 | 0.045 | Both sexes | Nucleotide phosphate-binding region: NAD, active site: Proton acceptor |
| B12 | 0.049 | Both sexes | Innate immune response, immunity |
| F1 | 0.024 | Females only | Oxidoreductase, oxidoreductase activity, oxidation-reduction process |
| F2 | 0.024 | Females only | Glycolytic process, glycolysis, biosynthesis of amino acids |
| M1 | 0.000 | Males only | Cytoplasmic translation, structural constituent of ribosome, ribosomal protein |
| M2 | 0.011 | Males only | Protein biosynthesis, translational initiation |
| M3 | 0.022 | Males only | Transmembrane transport, major facilitator superfamily domain |
| M4 | 0.037 | Males only | DEAD-box, Helicase, ATP-binding domain |
| M5 | 0.038 | Males only | Glutamine metabolic process, glutamine amidotransferase |
| M6 | 0.048 | Males only | ATP binding, nucleotide-binding |
| I1 | 0.027 | Interaction | Transmembrane transport, integral component of membrane |

**Table S3 | Genes both significantly associated with variation in population Ctmin (from (Wiberg *et al.*, 2020)) and differentially expressed in response to cold in males and females**

| Gene | *D. melanogaster* ortholog |
| --- | --- |
| augustus_masked-scaffold845-size43869-abinit-gene-0.1 | - |
| maker-scaffold328-size92609-snap-gene-0.40 | sl |
| maker-scaffold1540-size28586-snap-gene-0.11 | ORMDL |
| maker-scaffold811-size79825-augustus-gene-0.38 | nes |
| augustus_masked-scaffold79-size113372-abinit-gene-0.6 | Pus7 |
| maker-scaffold294-size82556-augustus-gene-0.42 | cue |
| maker-scaffold5-size95213-augustus-gene-0.37 | Cpr |
| maker-scaffold568-size93449-augustus-gene-0.44 | CG9119;CG32335 |
| maker-scaffold1332-size21654-snap-gene-0.11 | CG13308 |
| augustus_masked-scaffold602-size52133-abinit-gene-0.3 | GNBP3 |
| maker-scaffold24-size118282-snap-gene-0.53 | Tsr1 |
| augustus_masked-scaffold137-size142473-abinit-gene-1.2 | Glg1 |
| maker-scaffold124-size51034-snap-gene-0.13 | Nha2 |
| augustus_masked-scaffold99-size88673-abinit-gene-0.4 | CG3164 |
| maker-scaffold1268-size31467-augustus-gene-0.6 | CG15406 |
| maker-scaffold227-size60494-augustus-gene-0.20 | Fatp1 |
| snap_masked-scaffold202-size159099-abinit-gene-0.13 | La |
| maker-scaffold269-size166296-augustus-gene-1.22 | - |
| augustus_masked-scaffold845-size43869-abinit-gene-0.0 | Mdh1 |
| maker-scaffold2327-size32305-augustus-gene-0.7 | vri |
| maker-scaffold609-size49904-augustus-gene-0.24 | Tspo |
| augustus_masked-scaffold1173-size78255-abinit-gene-0.12 | mRpS23 |
| snap_masked-scaffold1317-size67552-abinit-gene-0.14 | hang |
| maker-scaffold1365-size77360-snap-gene-0.56 | Had1 |
| augustus_masked-scaffold438-size69364-abinit-gene-0.9 | CG9672 |
| augustus_masked-scaffold1326-size36164-abinit-gene-0.1 | anox |
| maker-scaffold1317-size67552-augustus-gene-0.29 | CDC50 |
| augustus_masked-scaffold720-size40553-abinit-gene-0.3 | mRpL22 |
| snap_masked-scaffold1760-size29917-abinit-gene-0.5 | CG5004 |
| augustus_masked-scaffold1790-size14554-abinit-gene-0.1 | CG2202 |
| maker-scaffold1093-size72171-augustus-gene-0.21 | - |
| maker-scaffold642-size61208-augustus-gene-0.12 | CG12338 |
| maker-scaffold2573-size15920-snap-gene-0.11 | CG12896;Prx2540-2;Prx2540-1 |
| maker-scaffold274-size124964-augustus-gene-1.18 | l(2)k09913 |
| augustus_masked-scaffold212-size112983-abinit-gene-1.3 | AGBE |
| augustus_masked-scaffold191-size139346-abinit-gene-0.6 | Idgf6 |
| maker-scaffold191-size139346-augustus-gene-1.6 | CG15617 |
| maker-scaffold1168-size26903-snap-gene-0.7 | stw |
| maker-scaffold845-size43869-snap-gene-0.11 | Ufd4 |
| maker-scaffold582-size48677-augustus-gene-0.13 | aralar1 |
| maker-scaffold1188-size47956-augustus-gene-0.15 | CG34454 |
| maker-scaffold115-size186799-augustus-gene-0.25 | UK114 |
| snap_masked-scaffold359-size110304-abinit-gene-1.3 | REPTOR |
| augustus_masked-scaffold195-size50053-abinit-gene-0.8 | Kyat |
| maker-scaffold363-size96300-augustus-gene-0.23 | CG1523 |
| maker-scaffold177-size153351-snap-gene-0.75 | CG8349 |
| maker-scaffold472-size153788-augustus-gene-0.32 | CG10208 |
| maker-scaffold1317-size67552-augustus-gene-0.33 | - |
| maker-scaffold1816-size40619-augustus-gene-0.3 | - |
| maker-scaffold2042-size28187-snap-gene-0.11 | - |
| maker-scaffold2042-size28187-snap-gene-0.13 | - |
| maker-scaffold2331-size31525-augustus-gene-0.6 | - |
| maker-scaffold2771-size22421-snap-gene-0.4 | - |
| maker-scaffold2946-size47827-snap-gene-0.1 | - |

**Table S4 | Accession numbers for sequenced samples**

| Accession number | Temperature | Sex | Replicate number |
| --- | --- | --- | --- |
| SRR10960345 | 6 | female | 1 |
| SRR10960337 | 6 | female | 2 |
| SRR10960346 | 6 | female | 3 |
| SRR10960342 | 19 | female | 1 |
| SRR10960341 | 19 | female | 2 |
| SRR10960343 | 19 | female | 3 |
| SRR10960339 | 6 | male | 1 |
| SRR10960340 | 6 | male | 2 |
| SRR10960338 | 6 | male | 3 |
| SRR10960348 | 19 | male | 1 |
| SRR10960347 | 19 | male | 2 |
| SRR10960344 | 19 | male | 3 |

**Fig. S1 |** Overlap of sex-biased genes in control (19 °C) or cold treated (6 °C) samples

**
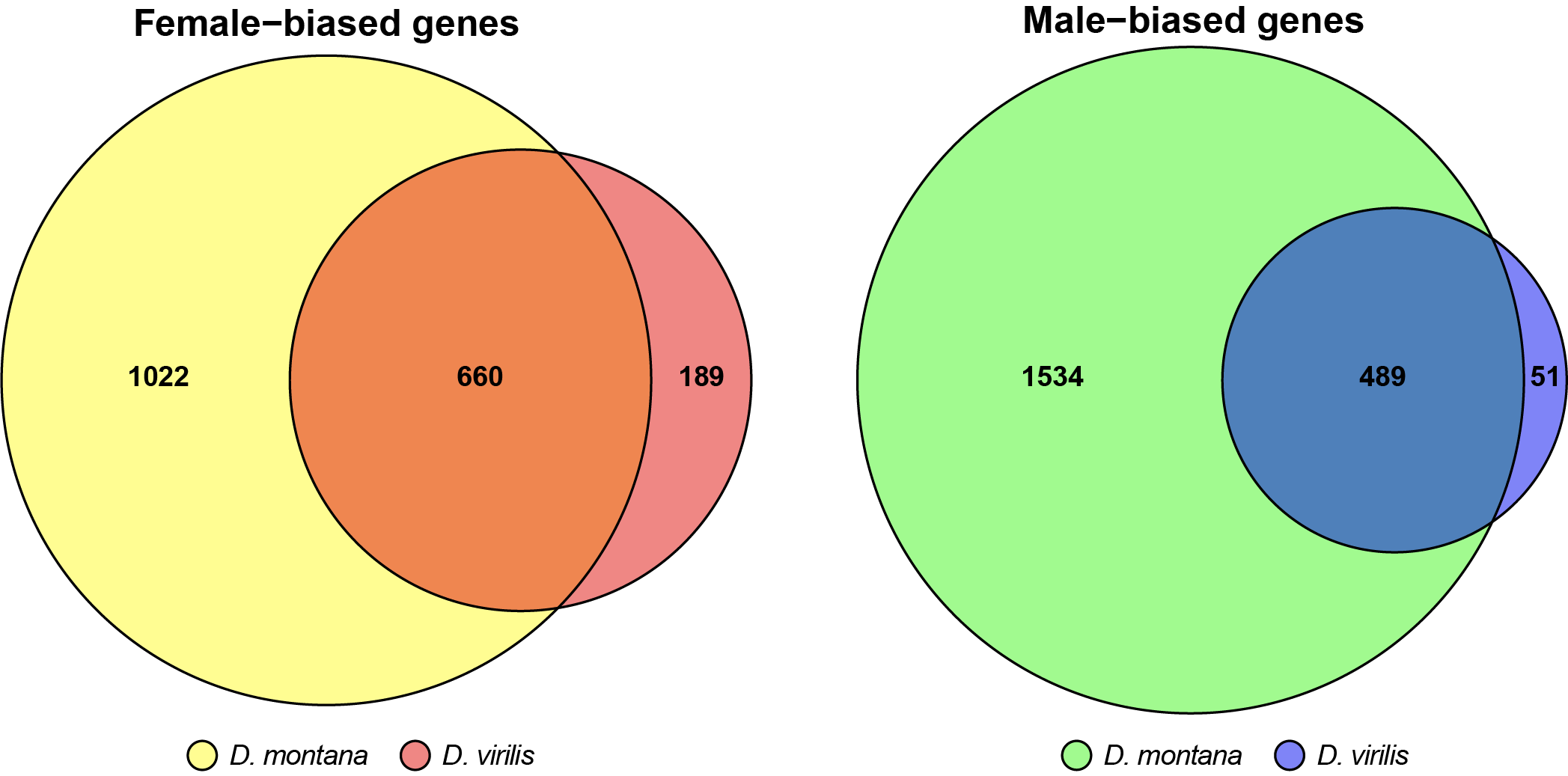
**

**Fig. S2 |** Overlap of sex-biased genes between *D. montana* and *D. virilis*

**
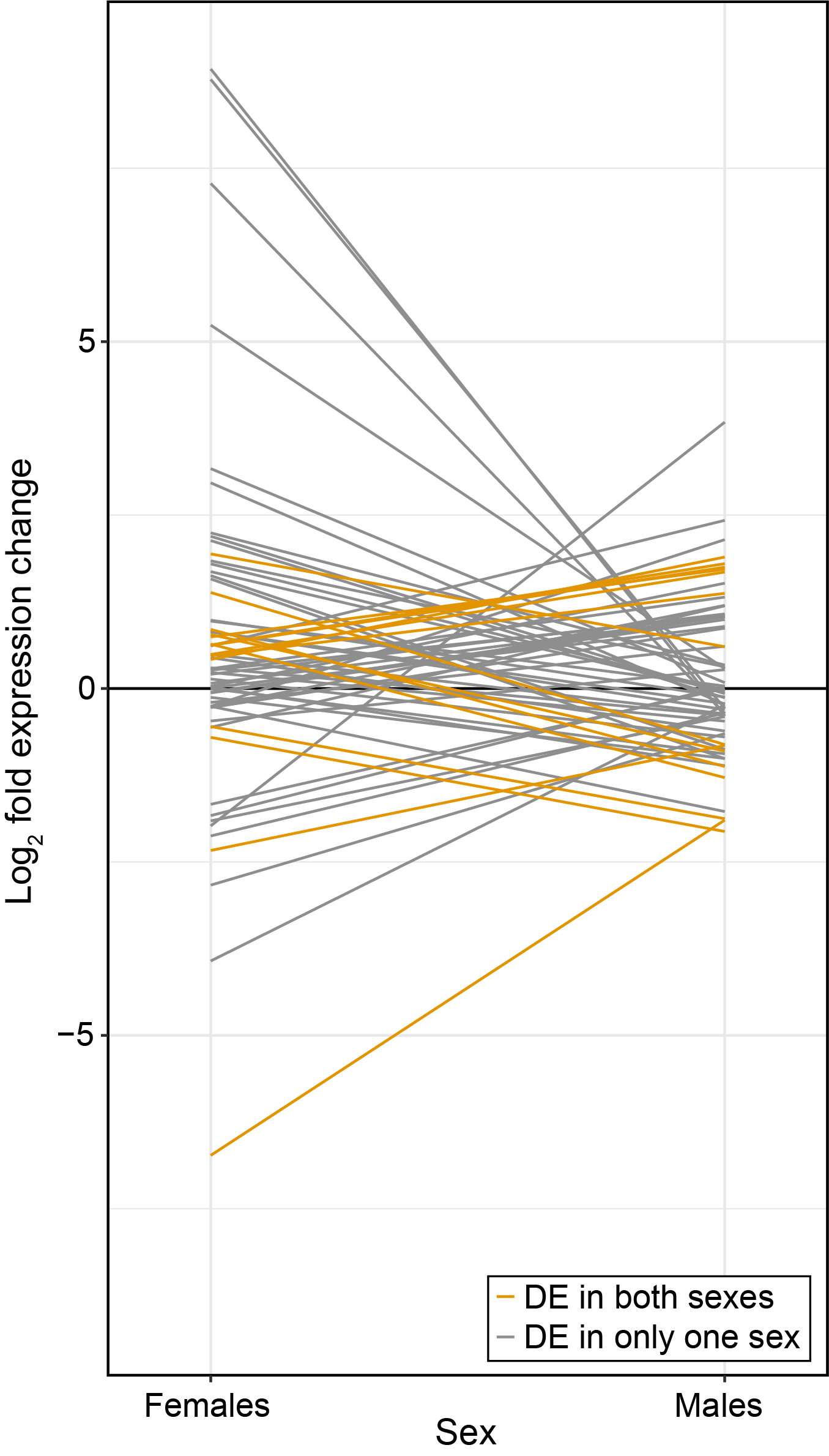

Fig. S3 |** Expression change in males and females for genes with a significant sex by treatment interaction**.** Orange = genes DE in both males and females. Grey = genes DE in either males or females.

**
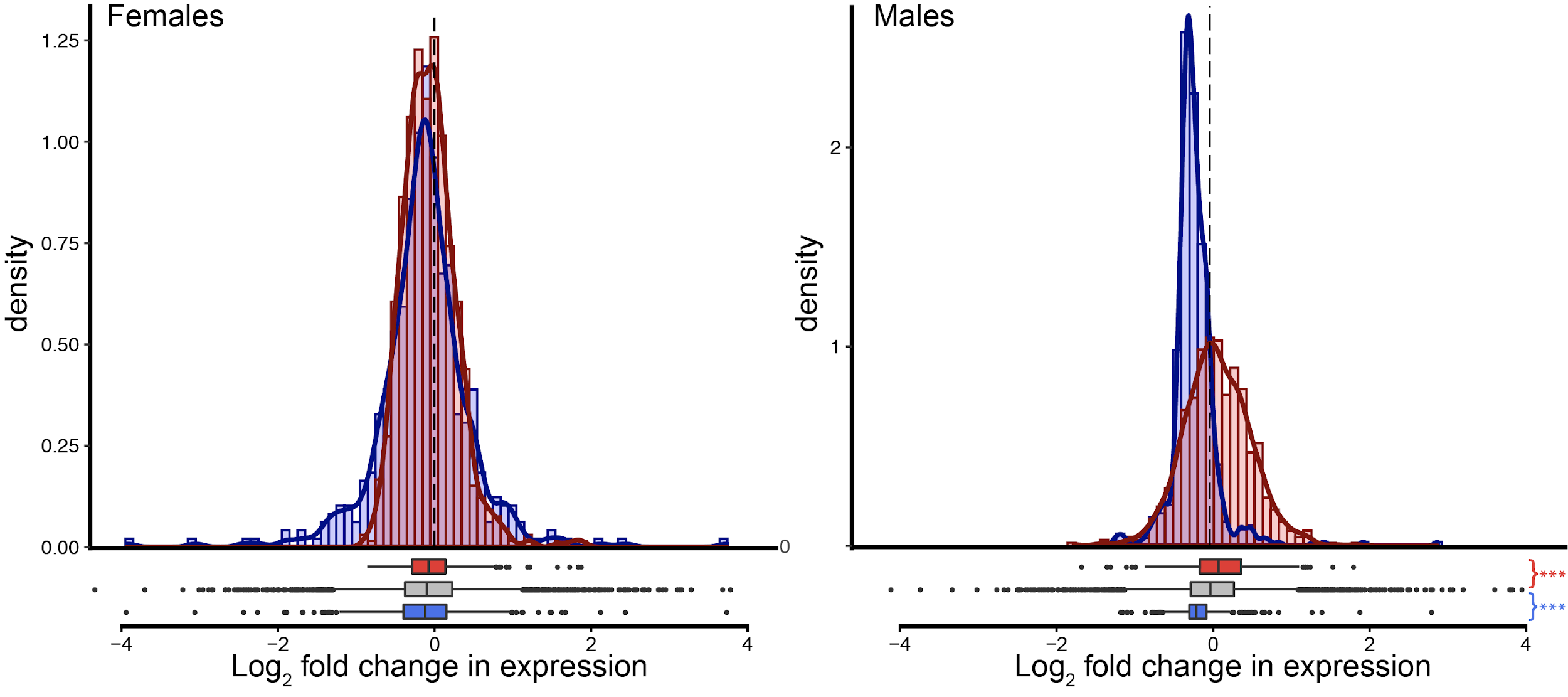
**

**Fig. S4 |** Expression shifts in genes sex-biased in both *D. montana* and *D. virilis* following cold treatment in females and males. Positive values indicate increased expression in cold-treated flies. Asterisks indicate the significance level (FDR) of Wilcoxon tests comparing the change in expression in female-biased (red, N = 660) and male-biased (blue, N = 489) genes to unbiased genes (***<0.001, **<0.01, *<0.05).


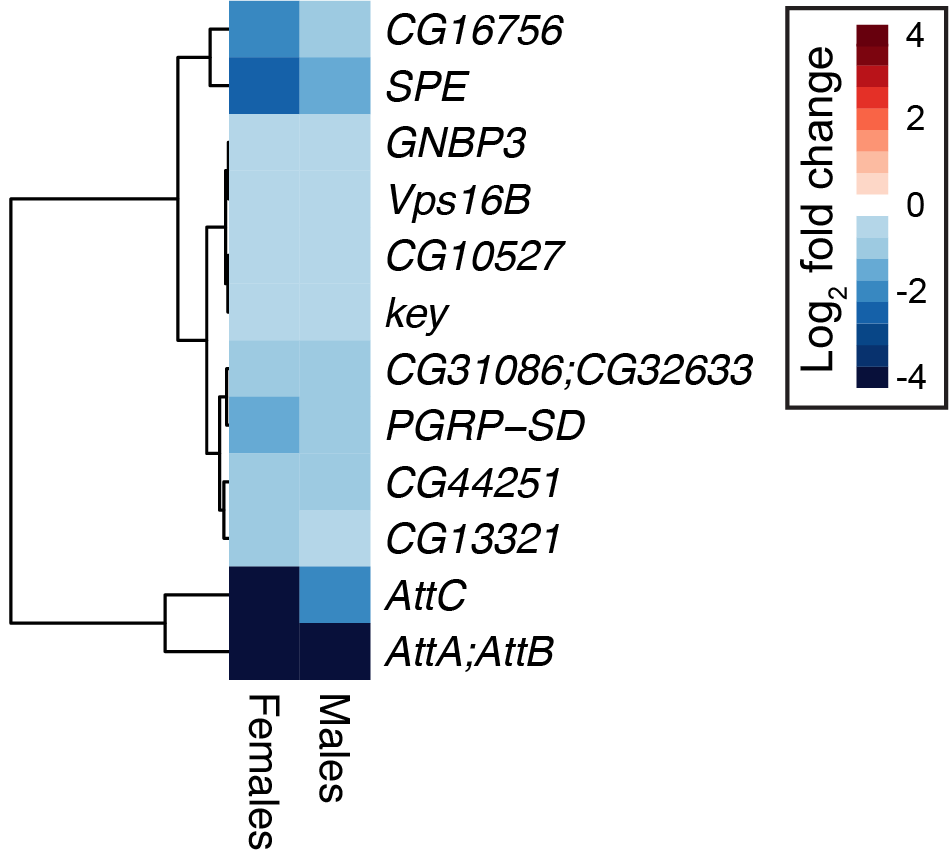


**Fig. S5 |** Expression shifts in genes enriched for innate immune response and DM9 repeats. Negative values indicate decreased expression in cold-treated flies.  **
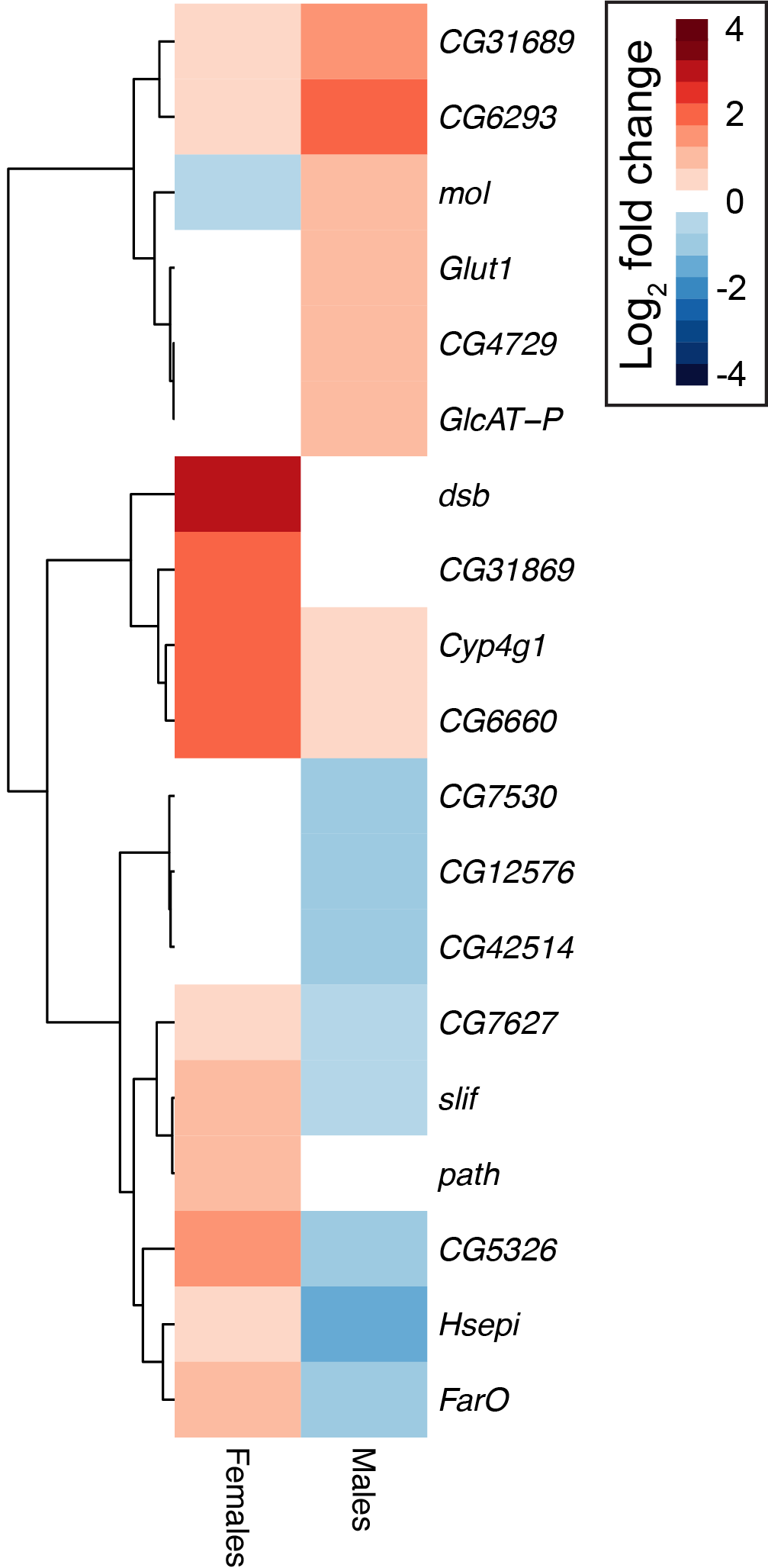

Fig. S6 |** Expression shifts in genes with a significant sex by treatment interaction enriched for transmembrane transport, integral component of membrane processes. Positive values indicate increased expression in cold-treated flies.

**
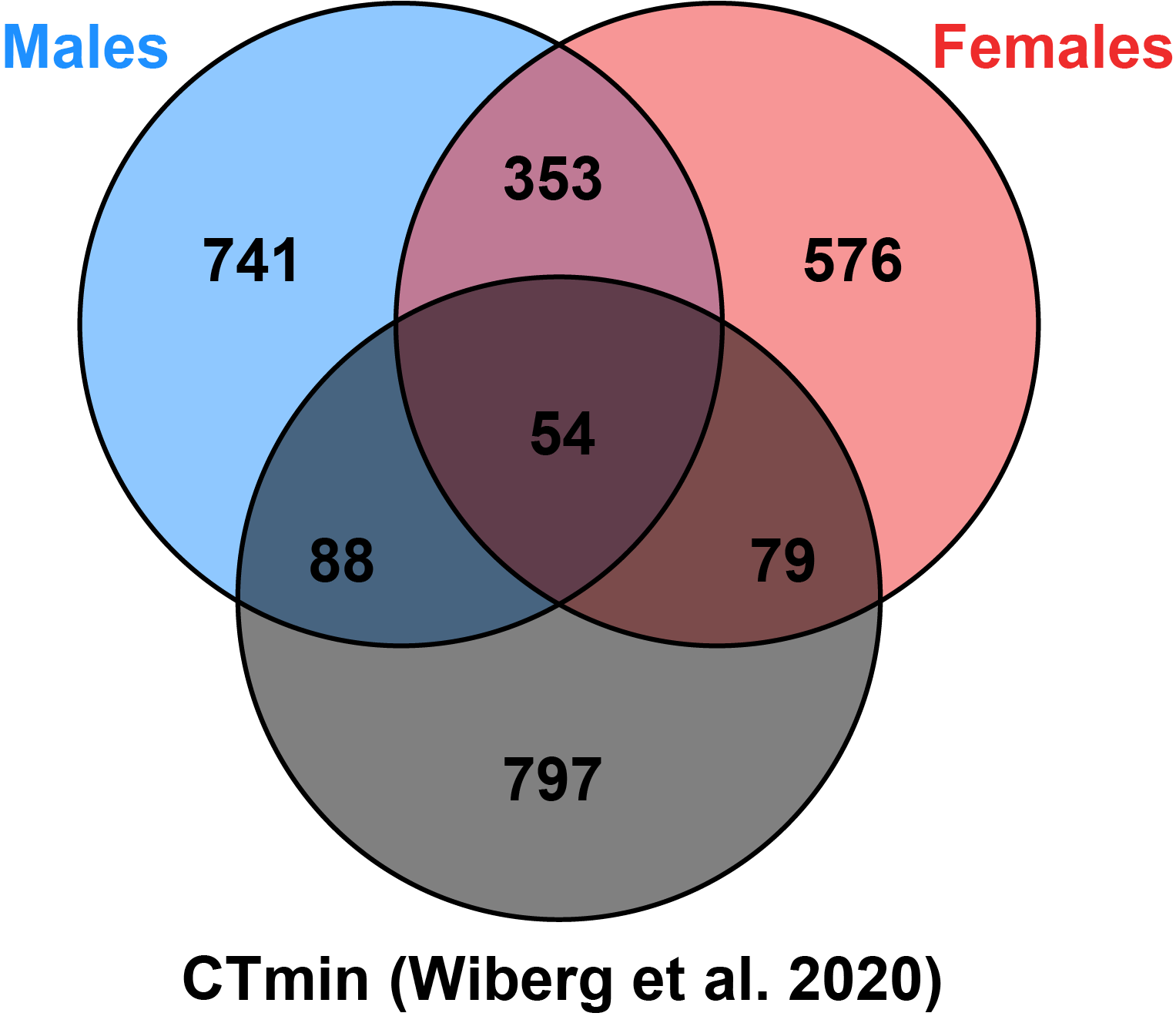
**

**Fig S7 |** Overlap between genes significantly associated with variation in population Ctmin (from Wiberg *et al.*, 2020, grey) and those differentially expressed in response to cold in males (blue) and females (red). The overlap between all three sets is greater than expected by chance (*p* = 1.93 x 10^-15^)

**References**

Wiberg RAW, Tyukmaeva V, Hoikkala A, Ritchie MG, Kankare M (2020). Cold adaptation drives population genomic divergence in the ecological specialist, *Drosophila* *montana.* *BioRxiv*: 2020.04.20.050450.
